# Microwave photon detection by an Al Josephson junction

**DOI:** 10.3762/bjnano.11.80

**Published:** 2020-06-23

**Authors:** Leonid S Revin, Andrey L Pankratov, Anna V Gordeeva, Anton A Yablokov, Igor V Rakut, Victor O Zbrozhek, Leonid S Kuzmin

**Affiliations:** 1 Institute for Physics of Microstructures of RAS, GSP-105, Nizhny Novgorod, 603950, Russia; 2 Center of Cryogenic Nanoelectronics, Nizhny Novgorod State Technical University, Nizhny Novgorod, Russia; 3 Lobachevsky State University of Nizhny Novgorod, Nizhny Novgorod, Russia; 4 Chalmers University of Technology, SE-41296 Gothenburg, Sweden

**Keywords:** aluminium, Josephson junction, microwaves, phase diffusion, photon counter, switching current distribution

## Abstract

An aluminium Josephson junction (JJ), with a critical current suppressed by a factor of three compared with the maximal value calculated from the gap, is experimentally investigated for application as a threshold detector for microwave photons. We present the preliminary results of measurements of the lifetime of the superconducting state and the probability of switching by a 9 GHz external signal. We found an anomalously large lifetime, not described by the Kramers’ theory for the escape time over a barrier under the influence of fluctuations. We explain it by the phase diffusion regime, which is evident from the temperature dependence of the switching current histograms. Therefore, phase diffusion allows for a significant improvement of the noise immunity of a device, radically decreasing the dark count rate, but it will also decrease the single-photon sensitivity of the considered threshold detector. Quantization of the switching probability tilt as a function of the signal attenuation for various bias currents through the JJ is observed, which resembles the differentiation between *N* and *N* + 1 photon absorption.

## Introduction

Currently, an important problem is the creation of single-photon counters in the gigahertz frequency range. Such devices are in demand in several areas, such as the search for axions, the alleged particles of dark matter [1–4] and quantum computing [5]. Commercially available single-photon detectors operate at frequencies of hundreds of terahertz and higher [6–7]. For the lower-frequency range, a new class of single-microwave-photon detectors is needed. With regard to this, a current-biased Josephson junction (JJ) is of particular interest for applications as a threshold detector since its phase dynamics is altered even by a weak probe field. Rich dynamics of the JJ constantly inspires new applications, such as thermometry [8–9], noise statistics [10–12] and single-photon detection [13].

There are, at least, two different approaches for the practical realization of single-photon detectors based on Josephson junctions, both having their advantages and disadvantages. The first approach relies on a continuous current sweep at a constant repetition rate and the measurements of the switching current distributions, from which the response and sensitivity can be determined [14–16]. In particular, in [16] the tunneling properties of a current-biased Josephson junction coupled with a resonator directly depend on the number of microwave photons in the resonator. The main disadvantages of this approach are the long initialization and freezing times of the detector. The detector works by slowly increasing the bias current from zero. This ramp takes seconds to avoid non-adiabatic excitation in a JJ. As soon as the detector switches, it must be reset by setting the current back to zero and waiting when a Josephson phase relaxes in a potential well. This implies a low repetition rate.

The second approach for experimental microwave detection [17–18] uses the switching events of a biased Josephson junction resulting from a single absorption. In contrast to the previous approach, this one requires less downtime of the detector, determined by the biasing time to the desired current only. However, the operation in this mode does not provide information on the number of absorbed photons and only above-threshold signals can be detected. Also, special care must be taken to minimize the false switching events of the detector due to thermal fluctuations and macroscopic quantum tunneling.

In this article the second approach is applied to a prototype of a single-photon counter described in [4]. We study the possibility of detecting photons in the gigahertz frequency range using an aluminium Josephson junction with a suppressed critical current. The main requirement to this counter is an extremely large lifetime (thousands of seconds), orders of magnitude larger than the switching time after the photon absorption (typically less than nanoseconds). In [4] it was shown theoretically that both the required sensitivity and the noise immunity can be reached at the same time in JJ with a suppressed critical current. Besides that, the mesoscopic junctions with low critical currents have received a great deal of interest themselves, since they exhibit Josephson phase diffusion [19–23].

The Josephson phase diffusion in small junctions has been studied both experimentally [24–25] and theoretically [26]. Recently, this regime has been observed also in layered high-temperature superconductors [27]. The significance of this effect depends on the ratio of thermal fluctuations *k*_B_*T*, the damping parameter α and the Josephson energy *E*_J_. Here we will consider a small tunnel junction with the thermal noise intensity of γ = *k*_B_*T*/*E*_J_ ≥ 2 × 10^−2^ and α *>* 0.1, and show experimentally an unusually large lifetime of the superconducting state, which we attribute to phase diffusion according to [20]. The increase of the lifetime of the superconducting state due to phase diffusion was also observed in [28] under similar conditions. However, phase diffusion is expected to decrease the sensitivity to single photons for the same reason that it improves the noise immunity. To our knowledge, to date there are no works dedicated to the role of phase diffusion in the response to single photons. In the last section of the article, we show the experimental results of the switching probability induced by a weak microwave signal and discuss some features of the measured response, which may indicate the sensitivity to several photon bunches.

The analysis of the phase-diffusion phenomena is a special case of a general problem of the motion of a Brownian particle in a washboard potential in the framework of the resistively and capacitively shunted junction (RCSJ) model for the dynamics of the Josephson phase [29–30]. The tilt of the washboard potential is controlled by the bias current *I* and is defined as *E*_J_(*I*/*I*_C_), where *I*_C_ is the critical current and *E*_J_ = ℏ*I*_C_/2*e*. The particle moves along the potential in the presence of friction, the strength of which is characterized by α = ω_p_/ω_c_, where 
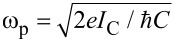
 is the plasma frequency, ω_c_ = 2*eI*_C_*R*_N_/ℏ is the characteristic frequency, *R*_N_ is the normal state resistance and C is the capacitance.

The superconducting state of the JJ corresponds to the particle at rest in one well of the potential. The exit from this metastable state corresponds to the appearance of a finite voltage at the junction. In the case of low damping (but depending also on the barrier height and noise intensity), the particle, jumping over the barrier, gains enough energy to move along the potential in the running state. When the damping α is sufficiently large, the escape due to the thermal or quantum fluctuations does not necessarily lead to the appearance of the running state. After an escape event, the particle can move down the potential for several wells and then relax into one of the potential minima [24]. When barrier and noise are large, the exit from the well and the subsequent retrapping processes may occur many times at a given bias current.

The most evident signature of the phase diffusion phenomenon is the temperature dependence of the switching current distribution [21,31]. For underdamped junctions (α ≪ 1), the width of the switching current distributions monotonically decreases with decreasing temperature. In the case of moderately damped junctions (α *>* 0.2), the switching dynamics changes due to phase diffusion, i.e., the width of the distribution decreases with increasing temperature. A change in the sign of the derivative of the second moment of the distribution is a reliable indicator of retrapping processes. Another sign of phase diffusion is an increase in the lifetime of the superconducting state in comparison with the classical Kramers’ theory [32–33]. The exit of the particle from the well due to fluctuations does not lead to the instantaneous appearance of a final voltage at the Josephson junction, which can be seen in experiment as an increase of the noise immunity of the system.

The principle of operation of a single-photon counter based on a Josephson junction is the following: At an initial moment of time, the junction is in a superconducting state with bias current *I* close to the critical current. In standby mode, there is no voltage at the junction. An incoming external signal from a photon (current oscillations) can change the state of the system by switching it to a resistive state with a finite resistance value. At the same time the detector may be triggered spontaneously due to thermal fluctuations in the classical region of temperatures and tunneling through the barrier in the quantum case [15,34].

## Experimental

**Figure 1:**
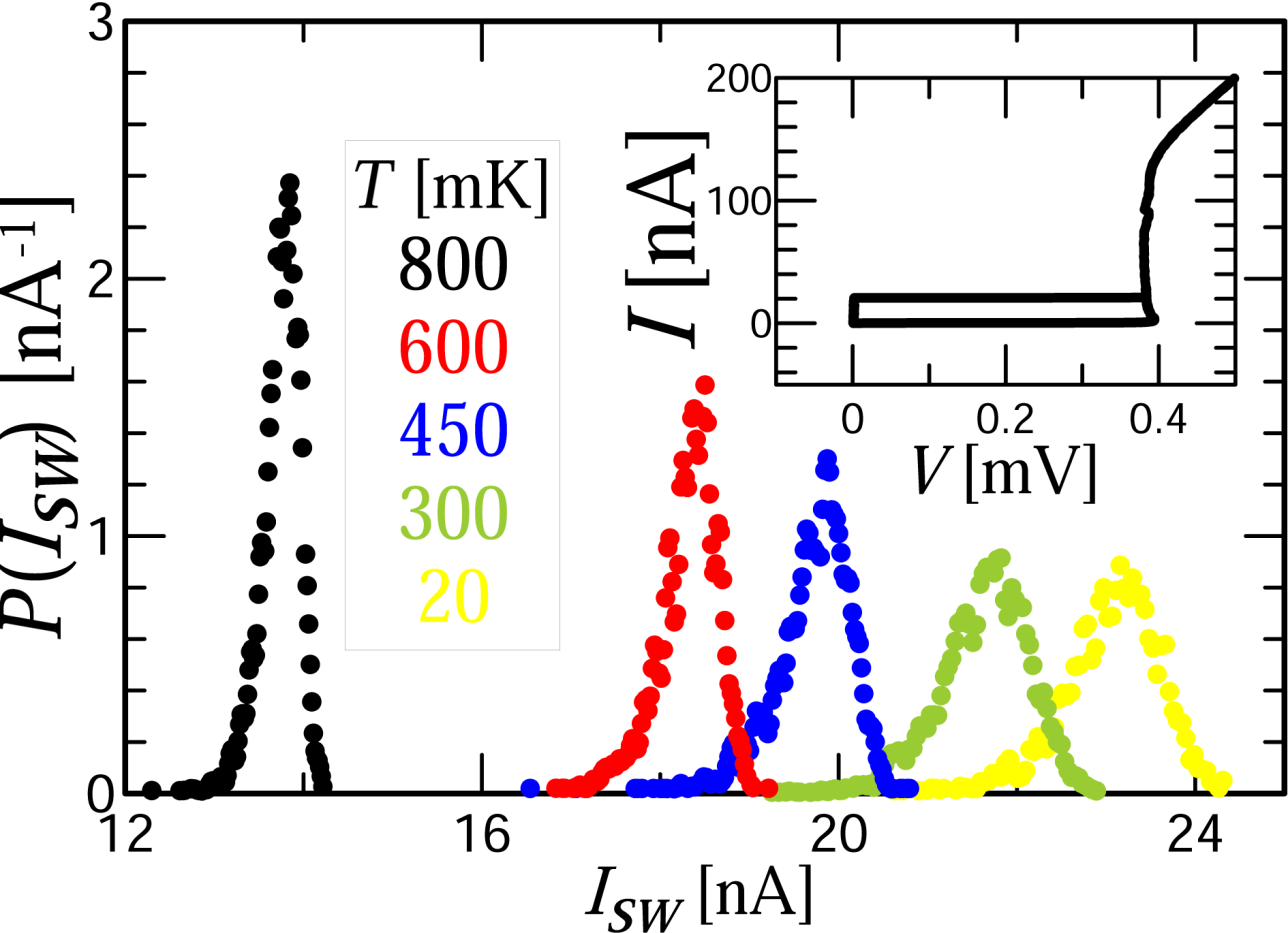
Experimentally measured histogram *P*(*I*_SW_) of switching the Josephson junction to the resistive state for the current *I*_SW_ at the indicated temperatures. The inset shows the *I*–*V* curve of the junction at 20 mK.

Following the line proposed in [4], an aluminium Al/AlO*_x_*/Al tunnel junction 0.4 × 2 µm^2^ was fabricated using a self-aligned shadow evaporation technique. Its current–voltage characteristic shown in the inset of Figure 1 (see below) has a well-defined hysteresis. The double voltage gap of the junction is 0.38 mV, corresponding to the critical temperature of Al, *T*_C_(Al) = 1.2 K, the capacitance is C ≈ 0.036 pF, the critical current density is 3.8 × 10^−3^ kA/cm^2^ and the normal resistance is *R*_N_ = 2300 Ω, which gives the maximal possible value of the critical current 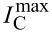 = 1.764 *kT*_C_/*eR*_N_ ≈ 80 nA. The measured critical current is *I*_C_ = 28 nA at a temperature of 20 mK. The damping of the Josephson junction calculated for the measured *I*_C_ is α = 0.24.

The sample was mounted in an rf-proof box with a superconducting shielding on the coldest plate of a Triton 200 dry dilution refrigerator. The dc-bias wires were filtered with feed-through capacitors at the room temperature and RC filters at the 10 mK cryostat plate, minimizing the effect of unwanted low-frequency noise. In order to avoid ground loops, the measurement scheme was designed with a single ground.

For the switching current measurements, the bias current of the junction was ramped up at a constant rate of 
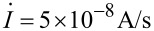
. The voltage was measured using a low-noise room-temperature differential amplifier AD745 and was fed to a high-speed NI ADC-card. This signal was used to trigger a fast record of the switching current value. This procedure was repeated at least 5 × 10^3^ times at each temperature, and as a result the switching current histograms were compiled in the temperature range between 1 K and 20 mK. For the lifetime measurements, the experimental setup was the same, except that the bias current was set to a predetermined value for 20 ms to prevent particle excitation caused by a rapid decrease in the barrier, and remained constant until the appearance of a gap voltage due to thermal noise or quantum tunneling. The lifetime measurements were repeated at least 200 times for each value of the bias current.

For a high-frequency experiment, a microwave signal was fed into the cryostat via a 2 m long phosphor bronze twisted-pair wiring with an attenuation of −15 dB/m at 9 GHz, and with a loop antenna near the JJ. The rf-signal from the external microwave synthesizer was attenuated using the voltage-controlled room-temperature attenuator, preliminarily calibrated with a commercial spectrum analyzer. The high-frequency signal was varied from a high power, at which the Shapiro steps and photon-assisted tunneling steps are well pronounced at the *I*–*V*-curve, to a low power, the presence of which can be observed only in the switching histograms and in the decrease of the superconducting state lifetime. While it is difficult to calibrate the whole rf path due to uncertainties in the twisted-pair wiring and the loop antenna, one can make estimates, based on the comparison of the potential barrier height of the JJ at 23 nA bias current and *I*_C_ = 28 nA (see the fit of the lifetime below). In this case, the potential barrier height is 1.3 × 10^−24^ J, while the photon energy at 9 GHz is 6 × 10^−24^ J. Thus, we are in the range where few-photon detection is possible.

## Results and Discussion

**Figure 2:**
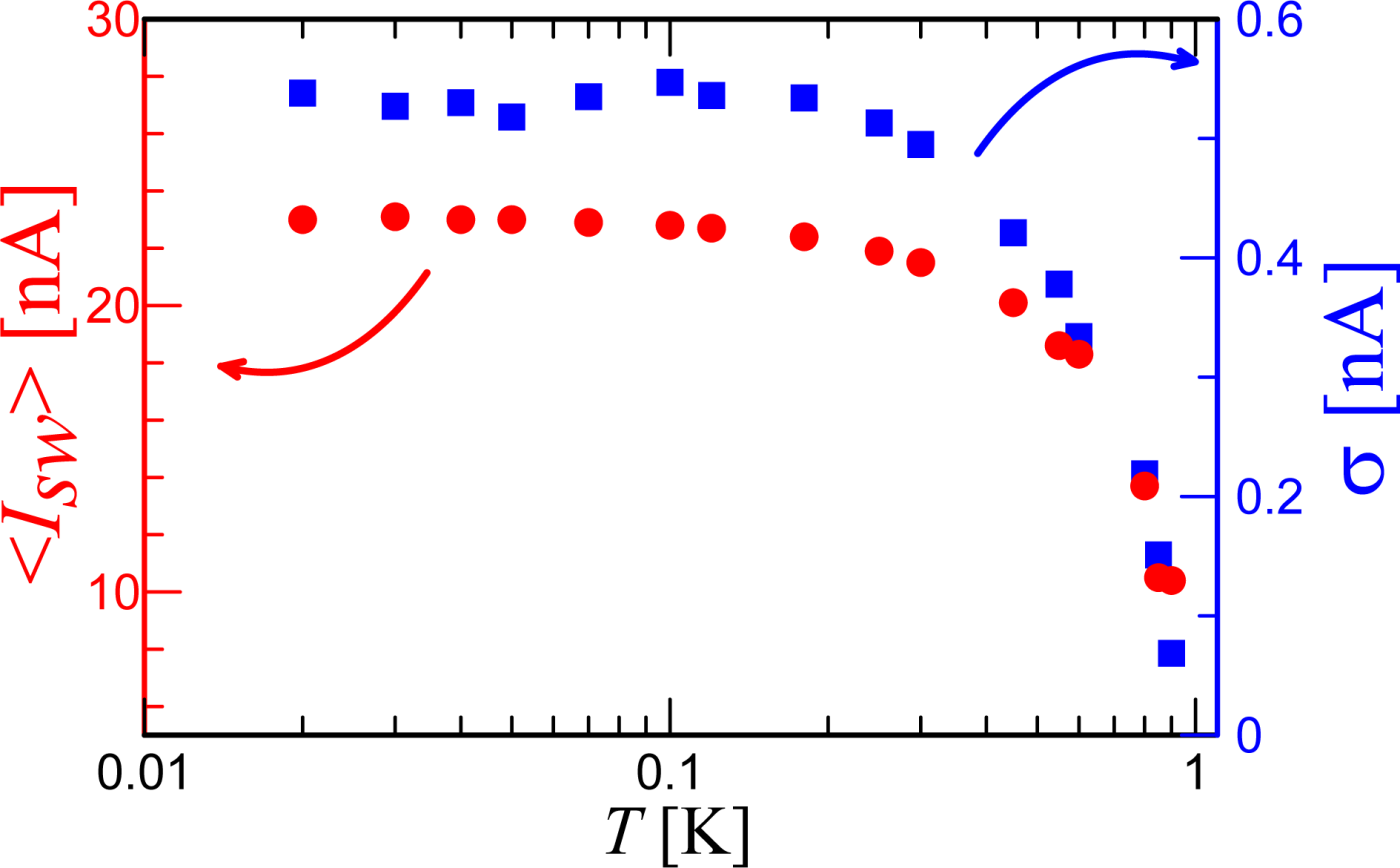
Temperature dependence of the mean switching current (left axis, red dots) and its standard deviation (right axis, blue squares).

In this section, we present preliminary results of the first measurements. First, we assemble the switching current distributions (Figure 1) and extract values for the mean switching current ⟨*I*_SW_⟩ and standard deviation σ, which are plotted in Figure 2 for different temperatures of the chip. The decrease of ⟨*I*_SW_⟩ with temperature increase indicates that here the thermal activation of the phase is the main switching mechanism. At temperatures below *T* ≈ 300 mK there is a saturation both in ⟨*I*_SW_⟩ and σ. The behavior of σ(*T*) in the entire temperature range of the experiment shows the well-known signature of phase diffusion, observed for example in [21,25,31].

**Figure 3:**
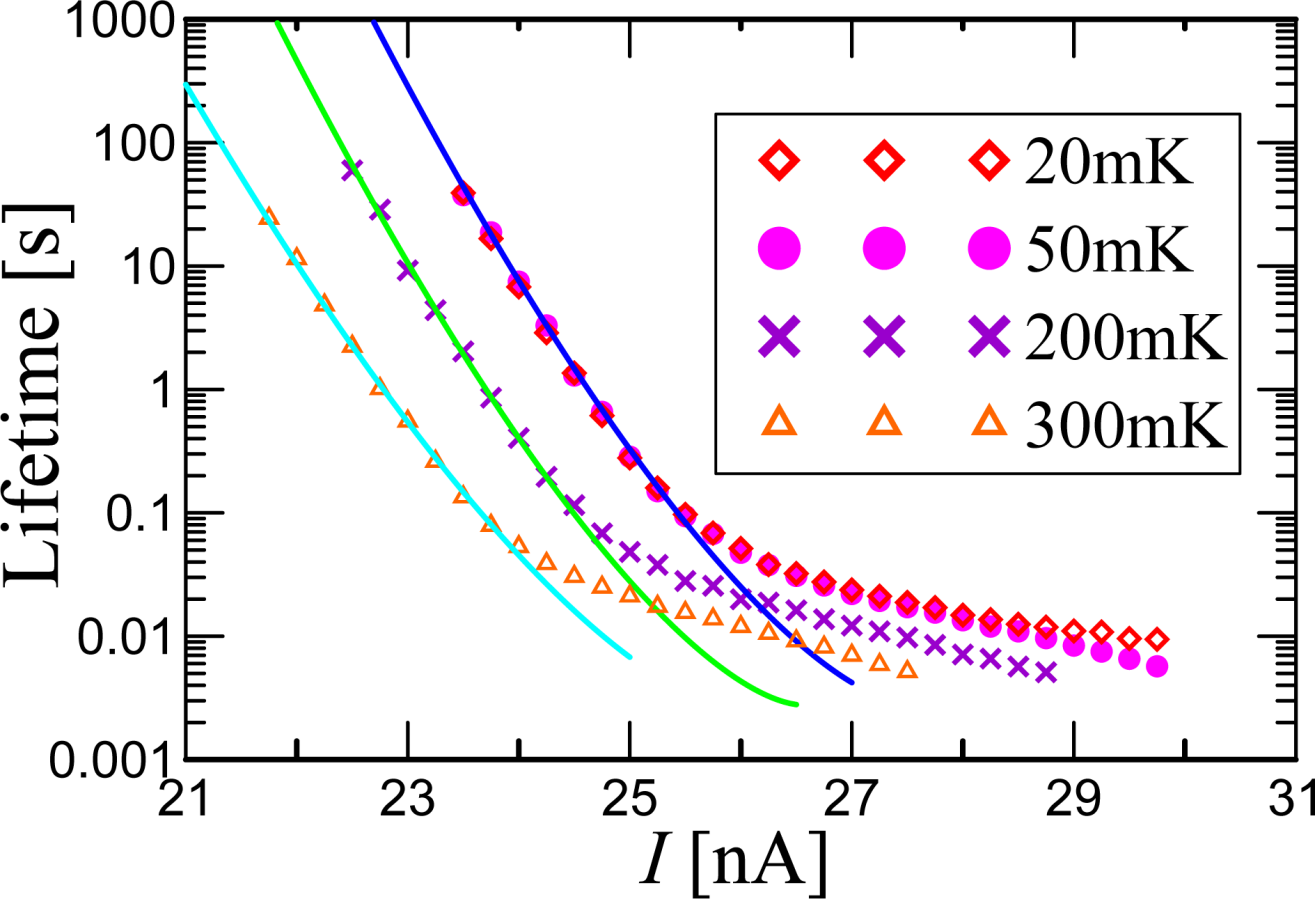
Experimental lifetime as function of the bias current for different sample temperatures (symbols) and fit with Equation 1 (solid curves).

The presence of phase diffusion can also explain the results of the lifetime (the inverse of the escape rate) measurements, shown in Figure 3. The lifetime of the superconducting state corresponds to the mean time of dark counts of a single-photon detector. We have measured the dependences of the lifetime for different bias currents and temperatures and without high-frequency signal. One can see the linear slope of the lifetime as a function of the bias current for 2–3 orders of magnitude on a logarithmic scale, which means the exponential dependence of the lifetime on the potential barrier height. The gentle slope of the experimental points, actually forming a plateau below 0.03 s in Figure 3, is due to time constants of the measurement setup. To find out more about the switching conditions the experimental curves have been fitted by the Kramers’ formula for the lifetime in the following form [30,32–33] (for the overdamped case, see [35]):

[1]
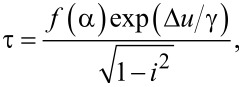


where *i* = *I*/*I*_C_ is the dimensionless bias current, 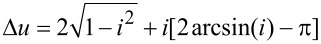 is the potential barrier height and γ = *I_T_*/*I*_C_ is the noise intensity and *I_T_* = 2*ek*_B_*T*/ℏ is the fluctuational current, which can be calculated for a given temperature *T* as *I_T_* [μA] = 0.042*T* [K] [29]. If the well and the barrier of a potential profile can be approximated by parabolas, then *f*(α) does not depend on the working temperature [36]. However, for α ≈ 1, the exact prefactor *f*(α) is unknown [33], therefore we use *f*(α) as a fit parameter.

Inserting a temperature of 300 mK into γ for our experimental parameters, one obtains γ = 0.48. For such large fluctuations the barrier height even with zero bias current is comparable to the noise intensity and the corresponding lifetime must be much smaller than measured in the experiment. If we use γ as a fit parameter together with *f*(α), we get the best fit for the following parameters: *f*(α) = 0.00035 s for all curves, *I*_C_ = 26.5, 27 and 28 nA, and noise intensity γ = 0.0137, 0.0112 and 0.011 for temperatures of 300, 200 and 50 mK, respectively. One can see that *I*_C_ in this case corresponds to the measured values.

**Figure 4:**
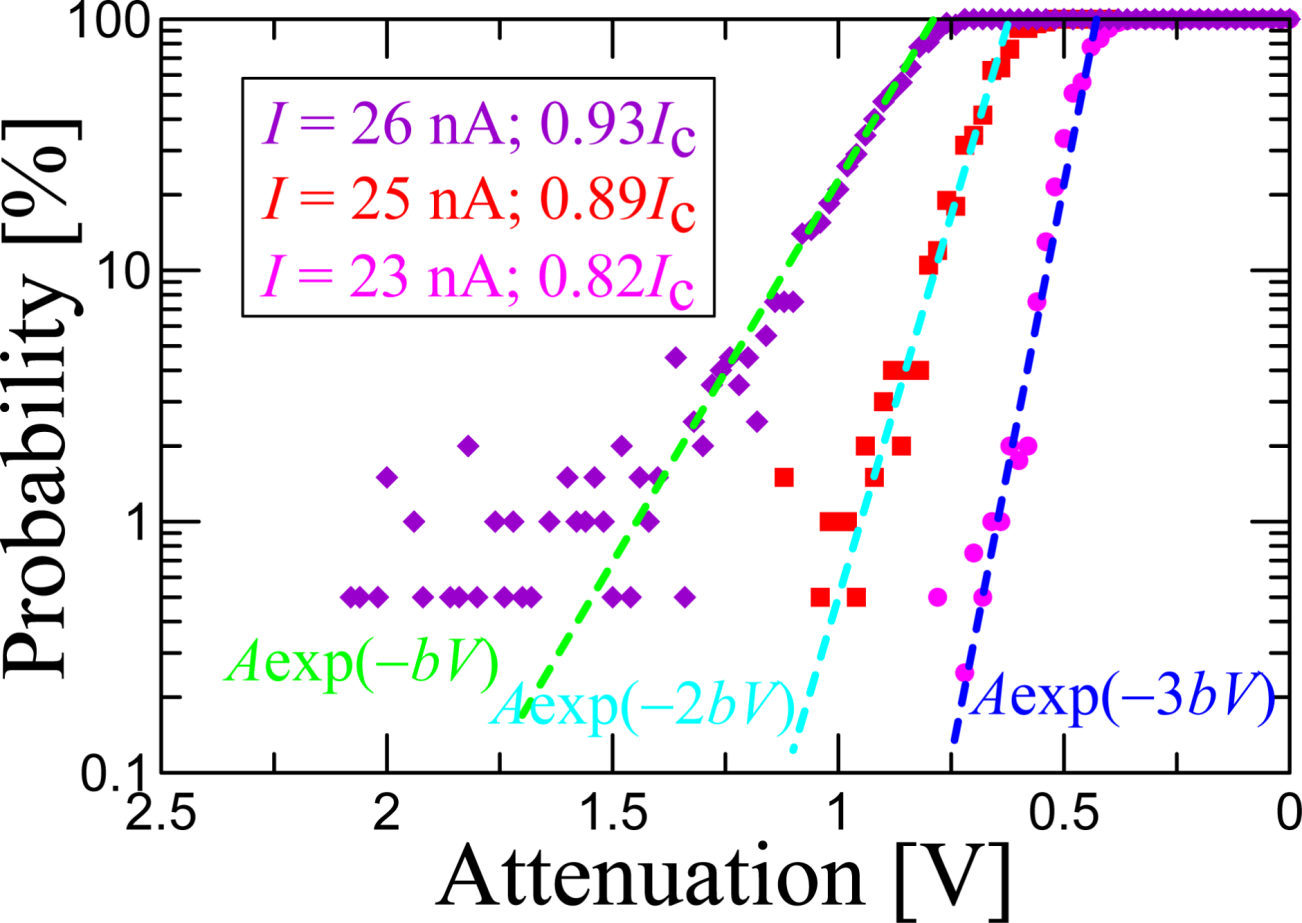
Detection probability of 9 GHz 50 ms pulses of different power (signal attenuation) for different values of the bias current. Dashed lines indicate slopes with exponential factors 1, 2 and 3.

Thus, the comparison of measurements and fit shows that the average time between dark counts significantly exceeds the time predicted by Kramers’ theory, with mean values reaching hundreds of seconds or even thousands of seconds in single measurements. Qualitatively similar discrepancies between experimental results and Kramers’ theory have been reported before [21,28] and require further studies. However, if it is the phase diffusion regime that significantly suppresses the dark count rate, the next important question will be to figure out how it influences the sensitivity to photons. In order to do so we perform measurements of the detection probability as a function of the attenuator voltage of 9 GHz photons in a 50 ms pulse, incident on the sample area, for three values of bias current *I*, shown in Figure 4.

The left vertical axis shows the experimental data, i.e., the number of detector counts divided by the total number of pulses (200 pulses). The horizontal axis corresponds to the attenuation (output power) of the external high-frequency signal. For high incident photon fluxes, the detector switches for all 200 pulses, i.e., it counts all pulses. For smaller fluxes our experimental data show that for 2.5 orders of magnitude, the detection probability decreases linearly (on a logarithmic scale) with the decrease of the incident power (average number of incident photons), and the probability slopes for various bias currents are well fitted by a function *A*·exp(−*nbV*), and are quantized. Here *A* and *b* are fit parameters and *b* is the same for all three curves. This resembles the multi-photon detection [6], where for a smaller bias current (*I* = 23 nA), the slope is larger, ca. *A*·exp(−3*bV*), than for larger bias current, ca. *A*·exp(−2*bV*) for *I* = 25 nA and ca. *A*·exp(−*bV*) for *I* = 26 nA.

Although we see a consistent switching due to 9 GHz signal even at 23 nA, at the moment we cannot estimate the absorption efficiency, because of the uncertainty in the determination of losses in the twisted-pair wiring at the frequency of 9 GHz and of the absorption efficiency in the junction. Therefore, we do not convert the attenuation to the power to avoid the introduction of an additional insecure parameter. The experiments will be continued with better statistics and signal calibration to extract the number of detected photons. We expect that the sensitivity of the considered threshold detector will be decreased in comparison with the situation without phase diffusion. However, further studies are required to answer this question.

## Conclusion

Temporal and detecting characteristics of a low-critical-current Al Josephson junction have been studied experimentally. From measurements of switching current distributions and the dark count time intervals, the operation in a phase diffusion regime is evident. It is shown by comparison with theory that the phase diffusion regime allows to significantly improve noise immunity of a device, radically increasing the mean time between dark counts. However, in the same way, the phase diffusion should decrease the single-photon sensitivity of the considered threshold detector, which will be studied in future experiments.

The plot of the detection probability as a function of the attenuation voltage shows quantization of the tail slopes, which resembles few-photon detection. The use of such a device for supersensitive detection has essential applications. In particular, such a detector can be used in the search for axions and to measure signals generated by quantum circuits at a frequency of 6–9 GHz. In the future, it is planned to improve the measurement setup and conduct research on the detection of test signals in the range of 8–14 GHz.
